# Allele-Specific Regulation of the Candidate Autism Liability Gene *RAI1* by the Enhancer Variant rs4925102 (*C/G*)

**DOI:** 10.3390/genes15040460

**Published:** 2024-04-06

**Authors:** Xi Yuan, Li Chen, David Saffen

**Affiliations:** 1 Institutes of Brain Science, Fudan University, Shanghai 200032, China; xyuan9209@163.com; 2 Department of Cellular and Genetic Medicine, School of Basic Medical Sciences, Fudan University, Shanghai 200032, China; 3 State Key Laboratory of Medical Neurobiology, Fudan University, Shanghai 200032, China

**Keywords:** regulatory variant, autism, autism spectrum disorders, *retinoic acid-induced 1* (*RAI1*), rs4925102, RARα/RXRα

## Abstract

*Retinoic acid-induced 1* (*RAI1*) is a dosage-sensitive gene that causes autistic phenotypes when deleted or duplicated. Observations from clinical cases and animal models also suggest that changes of *RAI1* expression levels contribute to autism. Previously, we used a bioinformatic approach to identify several single nucleotide polymorphisms (SNPs) located within the 5′-region of *RAI1* that correlate with *RAI1* mRNA expression in the human brain. In particular, the SNP rs4925102 was identified as a candidate *cis*-acting regulatory variant, the genotype of which may affect the binding of transcription factors that influence *RAI1* mRNA expression. In this study, we provide experimental evidence based on reporter gene, chromatin immunoprecipitation (ChIP), and chromatin conformation capture (3C) assays that rs4925102 regulates *RAI1* mRNA expression in an allele-specific manner in human cell lines, including the neuroblastoma-derived cell line SH-SY5Y. We also describe a statistically significant association between rs4925102 genotype and autism spectrum disorder (ASD) diagnosis in a case-control study and near-statistically significant association in an Autism Genome Project (AGP) transmission disequilibrium (TDT) study using Caucasian subjects.

## 1. Introduction

*Retinoic acid-induced 1* (*RAI1*) is a dosage-sensitive gene, for which a 50% change in expression levels due to deletion/mutation or duplication of a single copy causes Smith–Magenis syndrome (SMS) [1,2,3] or Potocki–Lupski syndrome (PTLS) [4], respectively. The occurrence of autistic phenotypes in clinical cases with SMS and PTLS has been reported to be over 60% (PTLS) [5] to over 90% (SMS) [6]. Shared abnormalities in the two syndromes, including intellectual disability and disorders of language, motor functions, and sleep, are also often observed in autism spectrum disorders (ASD) [7,8]. *RAI1* is not only the causal gene for a small fraction of patients with syndromic autism, but also a gene crucial for normal development of neural systems [9]. These observations strongly suggest that differences in *RAI1* expression caused by common regulatory genetic variants may contribute to autism and autistic symptoms.

Our previous results suggested that common genetic variants located in the 5′-region of the *RAI1* gene account for as much as 30–50% of the variance in *RAI1* expression in Chinese human brain samples [10]. Among these, the single nucleotide polymorphism (SNP) rs4925102 was identified as a likely functional regulatory variant that alone accounts for 24% (for dorsal lateral cortex) and 40% (for temporal cortex) of the variance in *RAI1* mRNA levels. Rs4925102 is located within the first intron of the human *RAI1* gene, about 12 Kb downstream from the transcription start site (TSS). According to information obtained via the UCSC Genome Browser, the DNA segment containing this SNP is a highly conserved, “active regulatory” DNA region characterized by high sensitivity to DNase I and marked extensive histone-3 lysine27 acetylation (H3K27Ac) [10]. According to the data in RegulomeDB [11,12], rs4925102 is predicted to locate within the binding motif of heterodimeric RARα/RXRα transcription factors.

Retinoic acid (RA) regulates the transcription of about a sixth of the human genome by binding to retinoic acid receptors (RARs) [13]. RARα/RXRα heterodimers bind to retinoic acid response elements (RAREs) that include a direct repeat (DR) of the motif 5′-PuG(G/T) TCA-3′ separated by one or more nucleotides: DR1, DR2, DR5, etc. [14]. Binding of all *trans*-retinoic acid (atRA), the most potent endogenous ligand of RARs, or its analogues, changes the conformation of RARα/RXRα heterodimers in such a way that inhibits the binding of co-inhibitors and stimulates the binding of co-activators [15]. RARs and RXRs are expressed in both developing and mature brains [16,17,18], and evidence from vitamin A deficiency (VAD) and gene knockout (KO) models indicate that RA signaling is involved in the cellular patterning in developing brain [17,19] and cognition-related activities such as long-term potentiation (LTP) and long-term depression (LTD) in the developing and mature brain [20,21,22]. VAD during critical periods of development has been linked to irreversible impairments on hippocampus function [23], and ASD-like phenotypes [24].

In this study, we used reporter gene, chromatin immunoprecipitation (ChIP), and chromatin conformation capture (3C) assays to demonstrate allele-specific regulation of *RAI1* gene expression by rs4925102 (*G/C*). We also demonstrated a near-statistically significant association between rs4925102 genotype and autism diagnosis in an Autism Genome Project (AGP) transmission disequilibrium test (TDT) study [25] and a statistically significant association in a case-control study based on AGP case data and control data from an unrelated schizophrenia study [26].

## 2. Materials and Methods

### 2.1. Cell Culture

Human HEK-293 (#GNHu18) and SH-SY5Y (#SCSP-5014) cells were purchased from the Chinese Academy of Sciences’ Cell Bank/Stem Cell Bank (Shanghai, China). HEK-293 cells were grown in DMEM (Gibco, Grand Island, NY, USA) containing 10% fetal bovine serum (FBS; Gibco, Grand Island, NY, USA). SH-SY5Y cells were grown in 1:1 mixture of MEM (Gibco, Grand Island, NY, USA) and F12 (Gibco, Grand Island, NY, USA) with 10% FBS, 2 mM L-alanyl-L-glutamine dipeptide (Invitrogen, Carlsbad, MA, USA), 1 mM sodium pyruvate (Invitrogen, Carlsbad, MA, USA), and 1 mM nonessential amino acids (Invitrogen, Carlsbad, MA, USA). All cells were cultured at 37 °C under 5% CO_2_ and the culture medium was changed every 2–3 days.

### 2.2. atRA Induction

All-*trans* retinoic acid (atRA; Sigma-Aldrich, St. Louis, MO, USA) was dissolved in dimethyl sulfoxide (DMSO; Sigma-Aldrich, St. Louis, MO, USA) and stored at −80 °C. In atRA induction experiments, the final concentration of atRA was 1 μM [10,27] and 0.01% (*v*/*v*) for DMSO, based on our pilot dose–response experiment (Figure S2).

### 2.3. Plasmid Construct

Plasmid pGL4.23 [luc2/minP] (Promega, Madison, WI, USA; GenBank: 904455) was digested with *Xho* I (NEB; Beverly, MA, USA) and Hind III (NEB; Beverly, MA, USA). The inserted DNA fragment based on the human DNA sequence containing the rs4925102 *C*- or *G*-allele (Table S1) was synthesized and purified by Generay Biotech Co., Ltd. (Shanghai, China). The DNA sequence of the final plasmid construct was verified by Sanger sequencing at Majorbio Bio-Pharm Technology Co., Ltd. (Shanghai, China).

### 2.4. Luciferase Reporter Assay

Luciferase reporter plasmids were co-transfected with the pRL-TK control plasmid (Promega, Madison, WI, USA; GenBank: dq904455) at a ratio of 20:1 (200 ng/well and 10 ng/well, respectively) into HEK-29 using Lipofectamine 2000 (Invitrogen, Carlsbad, MA, USA). The luminescent signals were detected using a GloMax^®^ 20/20 Luminometer (Promega, Madison, WI, USA) or Synergy2 (BioTek, Winooski, VT, USA) 24 h after transfection. For experiments conducted with SH-SY5Y cells, only luciferase reporter plasmids were transfected into cells and total cell protein concentrations quantified by BCA protein assays (Pierce, Rockford, IL, USA) were used for normalization of the luciferase-catalyzed fluorescence.

### 2.5. Chromatin Immunoprecipitation (ChIP) Assays

ChIP assays were performed as previously described [10] with a few modifications as follows: an amount of 5 μg of each antibody (RARα; Abcam, Cambridge, MA, USA; RXRα; Sigma-Aldrich, St. Louis, MO, USA) was used for immunoprecipitation and a reaction containing no antibody (NoAb) was used as a negative control. Primer sequences to amplify the segment (176 bp) containing rs4925102 from purified DNA were: 5′-GGGCAGCCTTCCTGATTGACA-3′ (forward) and 5′-CCTGACCTTCGACAATGGCTT-3′ (reverse). The locations of these primers in relation to rs49205102 are shown in Figure S4c in Supplementary Materials. Images of immunoprecipitated DNA bands were obtained from raw images of entire electrophoretic gels uniformly processed to increase contrast (+40% for replicate 1) and brightness (+40% for both replications) using Microsoft Power Point 2021.

### 2.6. Quantification of Immunoprecipitated Chromatin Containing rs4925102 C- or G-Alleles

SH-SY5Y cells are heterozygous (*G/C*) for rs4925102 (Figure S3a). To quantify allele-specific binding to rs4925102, PCR-amplified DNA from chromosomal segments immunoprecipitated using transcription factor-specific antibodies was purified for Sanger sequencing (Majorbio Bio-Pharm Technology Co., Ltd.; Shanghai, China) and followed by the quantification of the relative abundance of the *C*- and *G*-alleles based on the areas under the relevant peaks in DNA sequencing electropherograms using image J v1.54g.

To compensate for intrinsic and context-specific differences in *C*- and *G*-allele-related fluorescence signals, sequencing electropherograms obtained with purified genomic DNA from SH-SH5Y cells were used to normalize the ratio of peak areas of the *C*-allele to *G*-allele of rs4925102 when sequencing immunoprecipitated DNA of unknown allelic composition. Given that equal amounts of *C*- and *G*-allele are expected in the genomic DNA isolated from SH-SY5Y cells, we defined index ***A*** as a correction factor that satisfies the equations as follows:

$$A_{Forward}\cdot \frac{C_{gDNA}}{G_{gDNA}}=1,$$

where *C_gDNA_* (or *G_gDNA_*) represents the area under the *C*- or *G*-peak of rs4925102 in the Sanger sequencing electropherograms obtained using PCR-amplified DNA produced from SH-SY5Y genomic DNA with forward primer. Three independent replicate experiments were performed to calculate the value of index ***A*** (Figure S3c). The non-normalized percentage of the rs4925102 *C*-allele (*C*%) in the immunoprecipitated DNA of unknown *C*-and *G*-allele composition varies as follows:

$$C\%=\frac{C_{sample}}{C_{sample}+G_{sample}}=\frac{\frac{C_{sample}}{G_{sample}}}{\frac{C_{sample}}{G_{sample}}+1},$$

Because of this the normalized ratios of the *C*-allele detected using the forward primer were calculated based on the equation below:

$${C\%}_{normalized}=\frac{A_{Forward}\cdot \frac{C_{sample}}{G_{sample}}}{A_{Forward}\cdot \frac{C_{sample}}{G_{sample}}+1}.$$

When using the reverse primer, the calculation is similar, except that the correction factor satisfies the following equation:

$$A_{Reverse}\cdot \frac{G_{gDNA}}{C_{gDNA}}=1,$$

where *G_gDNA_* (or *C_gDNA_*) represents the observed area under the *G*- or *C*-peak of rs4925102 in the Sanger sequencing electropherograms. See Figure S3 in Supplementary Materials for details concerning the measurement and calculation of ***A****_Forward_* and ***A****_Reverse_* correction factors.

### 2.7. Chromatin Conformation Capture, 3C

SH-SY5Y cells (1 × 10^7^) treated with 1 μM atRA or vehicle (0.01% DMSO) for 2 h were collected for chromatin conformation capture assays as previously described [28], with a few modifications. Briefly, cells were fixed at room temperature for 10 min by adding the appropriate volume of 16% paraformaldehyde solution (dissolved in PBS) to obtain a 1% final concentration, after which 0.125 M glycine was added to block further cross-linking. The cells were washed twice with cold PBS and briefly exposed to trypsin–EDTA (0.25%; Gibco, Grand Island, NY, USA) to facilitate collection. Cellular nuclei were released by exposing the cells to lysis buffer (5 mM PIPES, 85 mM KCl, 0.5% NP-40, pH 8.0) for 10 min on ice, concentrated by centrifugation (3000 rpm for 5 min) and stored at −80 °C for future use. Restriction enzyme Hind III (NEB; Beverly, MA, USA) and T4 DNA ligase (NEB; Beverly, MA, USA) were used to digest and ligate DNA, respectively, and for quality control purposes, including assessments of digestion efficiency and DNA purity, small aliquots of reactions before digestion, after digestion and after ligation were sampled and labeled UND (undigested), D (digested), or L (ligated), respectively. The control template used to optimize real-time PCRs was formed using a set of mixed constructions, that covered several restriction enzyme Hind III recognition sites within the gene *RAI1* region (Figure S5), in equimolar amounts. This series of DNA constructs was prepared by PCR-amplification following TA cloning (a simple method for the cloning of PCR products) [29] and verified by Sanger-sequencing (Majorbio Bio-Pharm Technology Co., Ltd.; Shanghai, China). The relative abundances of linkage products were quantified by touchdown quantitative PCR using the SYBR^®^ Green Realtime PCR Master Mix (TOYOBO, Kita-ku, Osaka, Japan) for best efficiency. Sequences of DNA primers used in these experiments are listed in Table S2.

PCR products were purified for Sanger sequencing (Majorbio Bio-Pharm Technology Co., Ltd., Shanghai, China), and the relative abundances of different SNP genotypes in the linked products were calculated based on areas under the relevant peaks in sequencing electropherograms using image J v1.54g. Calculations of *C*-allele abundance (*C*%) followed the method described in Section 2.6 above.

### 2.8. Statistics

Statistical analyses were performed using GraphPad Prism v9.0.0. Two-tailed Student’s *t*-tests were used for paired samples. One-way ANOVA followed by Tukey’s multiple comparisons test were used for comparisons of more than two conditions. Two-way ANOVA followed by Dunnett’s multiple comparisons were used for experiments with more than one group under different treatment as described in the figure legends. The results are represented by mean ± standard error of the mean (mean ± SEM), and the sample size n indicates the number of biological repetitions. *: *p* < 0.05; **: *p* < 0.01; ***: *p* < 0.001; ****: *p* < 0.0001; n.s., not significant: *p* > 0.05.

### 2.9. Samples

Phenotype and genotype data from the Autism Genome Project (AGP) [25] were obtained from dbGaP (Study Accession: phs000267.v1.p1). A TDT study comprising 4076 European individuals with probands diagnosed with autism spectrum disorder (phenotype ID: phv000267.v2.p2) was investigated.

Phenotype and genotype data from the NIH schizophrenia Genome Project [26] were obtained from dbGaP (Study Accession: phg000013.v1.p1). A number of 1298 European control individuals in this study and 1179 cases in the AGP TDT study were combined to carry out a case-control study.

### 2.10. Imputation

Imputation of human *RAI1* genotypes was carried out using Impute 2.0 with genotype data from the 1000 Genomes Project (Phase 1) as the reference. All imputed SNPs met the quality control (QC) criteria: missing rate < 0.05; SNP minor allele frequency > 0.01; *p*-value for deviance from Hardy–Weinberg equilibrium (HWE) > 0.001.

### 2.11. Association Analysis

SNP genotype-disorder associations in AGP trio samples were investigated using the transmission disequilibrium test (TDT) in PLINK. Our case-control study was carried out using case samples in the AGP trio study [25] and control samples in a schizophrenia case-control study [26]. Population structure was analyzed using Structure v2.3 software (http://pritch.bsd.uchicago.edu/structure.html (accessed on 29 September 2012)). Putative ASD risk alleles were identified based on the observed allelic odds ratios (ORs). The 95% confidence interval (95% CI) of ORs and the *p*-value were calculated using PLINK.

## 3. Results

### 3.1. Gene Expression Is Regulated by SNP Rs4925102 in an Allele-Specific Manner

**Figure 1:**
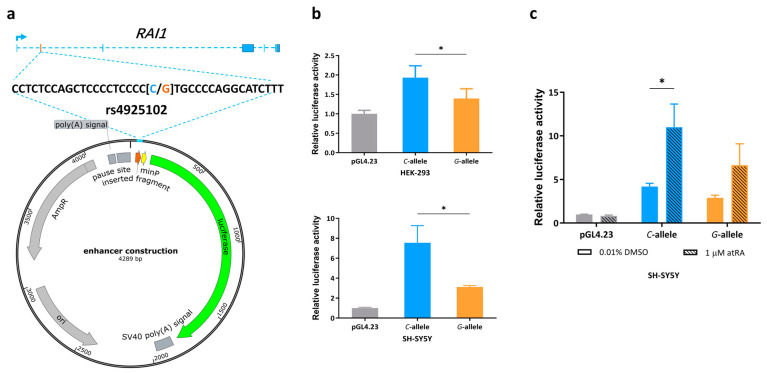
Evidence for transcription regulation activity of rs4925102 and allelic effects of rs4925102 on the enhancement of luciferase reporter gene expression by all-*trans* retinoic acid (atRA), (**a**) A 37 bp DNA fragment containing the SNP rs4925102 was inserted into the polyclonal site of the plasmid pGL4.23, which includes the coding sequence of a luciferase reporter gene under transcriptional control by a minimal promoter. (**b**) Luciferase activity was measured 24 h after transient transfection of this enhancer–reporter construct into two human cell lines, HEK-293 (human embryonic kidney cells) and SH-SY5Y (human neuroblastoma cells). Compared with the control plasmid, enhancer constructs containing either the rs4925102 *C-* or *G*-allele showed increased transcriptional activity, with expression under the presence of the *C*-allele significantly higher than the *G*-allele (*n* = 3 biological replicates). Data are presented as mean ± SEM. (*: *p* < 0.05, using one-way ANOVA and Tukey’s multiple comparison tests.) (**c**) SH-SY5Y cells were transfected with the enhancer constructs described in (**a**) and exposed to 1 μM atRA or 0.01% DMSO for 24 h prior to measurements of relative luciferase activity normalized to total cell protein (*n* = 3 biological replicates). Data are presented as mean ± SEM. (*: *p* < 0.05, based on two-way ANOVA and Sidak’s multiple comparison tests).

Using a luciferase reporter gene construct that includes SNP rs4925102(*G/C*) within a 37 bp human DNA segment inserted upstream of a minimal promoter (Figure 1a), we verified enhanced reporter gene expression in HEK-293 and SH-SY5Y cell lines (Figure 1b). Consistent with previous results obtained using human brain samples [10], reporter gene expression was higher in constructs containing the rs4925102 *C*-allele compared to *G*-allele in both cell lines.

### 3.2. Allelic Effects of Rs4925102 on Luciferase Reporter Gene Expression by All-Trans Retinoic Acid (atRA)

Because rs4925102 is predicted to bind RARα/RXRα heterodimers (Figure S1) [10], we hypothesized that atRA may increase *RAI1* expression via rs4925102. To test this idea, luciferase assays were carried out using SH-5H5Y cells transfected with the reporter plasmid described above following exposure of the cells to 1 μM atRA or 0.01% DMSO (vehicle control) for 24 h. As shown in Figure 1c, constructs containing the rs4925102 *C*-allele showed significantly increased transcriptional activity following exposure to atRA.

### 3.3. Preference of RARα/RXRα Binding Switches from the G- to C-Allele in the Presence of 1 μM atRA

**Figure 2:**
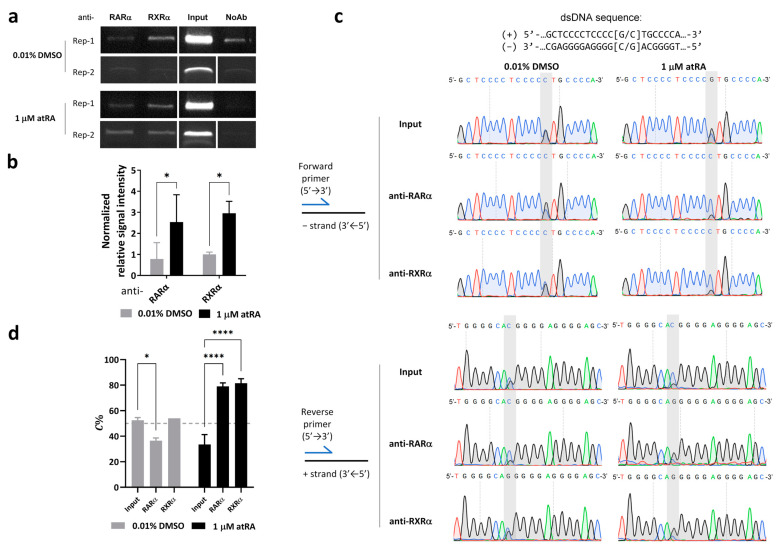
Preference of RARα/RXRα binding switches from *G*- to *C*-allele in the presence of 1 μM atRA. (**a**) Results for duplicate ChIP analyses using SH-SY5Y cells treated with 1 μM atRA or 0.01% DMSO for 2 h prior to immunoprecipitation of the nuclear extracts with antibodies specific for RARα or RXRα. As described in Section 2. Materials and Methods, enhancements of contrast (40% for replicate 1) and brightness (40% for both replications) were applied uniformly to raw images of entire gels using Microsoft Power Point software 2021 and the resulting images of bands from two biological replications were aligned together in the figure. (**b**) Quantification of immunoprecipitation assay results is shown in (**a**). Immunoprecipitation specificity was verified using no antibody (NoAb) controls. The relative intensities of immunoprecipitated chromosomal DNAs were calculated by subtracting the value obtained in the NoAb samples and then normalizing those corrected values with respect to the corrected value obtained for DNA bands immunoprecipitated with RXRα antibodies from control cells exposed to 0.01% DMSO. RARα and RXRα immunoprecipitations were enhanced in nuclear extracts prepared from cells exposed to atRA. (*: *p* < 0.05; based on two-factor analyses of variance and Sidak’s multiple comparison tests). (**c**,**d**) SH-SY5Y cells are heterozygous (*G*/*C*) for rs4925102. Sanger sequencing was used to detect the relative abundances of the *C*-and *G*-alleles in the immunoprecipitated chromatin. Representative Sanger sequencing electropherograms are shown in (**c**). (*C* = blue, *G* = black, *T* = red and *A* = green traces). Sequences of the + and − strands of the DNA fragment shown in the Sanger sequencing electropherograms in (**c**). are: GCTCCCCTCCCC [G/C]TGCCCA (+ strand, 5′- > 3′) and its complement TGGGCA [G/C]GGGGAGGGGAGC (− strand, 5′- > 3′). The quantitative results shown in (**d**) revealed that the proportion of *C*-allele (*C*%) in chromatin immunoprecipitated using antibodies specific for RARα or RXRα increased following 2 h exposure to 1 μM atRA (*: *p* < 0.05, ****: *p* < 0.0001; based on two-factor analyses of variance and Dunnett’s multiple comparison tests).

To determine the effects of atRA treatment on transcription factors binding to the rs4925102 DNA region, chromatin immunoprecipitation (ChIP) assays were carried out using nuclear extracts from SH-SY5Y cells exposed to 1 μM atRA or 0.01% DMSO (vehicle control) for 2 h (Figure 2a). SH-SY5Y cells were chosen for these studies because they are heterozygous for the rs4925105 *C*- and *G*-alleles. Quantification of agarose gel electrophoresis of two representative results showed that levels of RARα and RXRα binding in 1 μM atRA-treated cells are significantly higher than those in control cells (0.01% DMSO) (Figure 2b).

To quantify allele-specific binding to rs4925102, DNA immunoprecipitated with different transcription factors was PCR-amplified and the relative abundance of rs4925102 *C*- and *G*-alleles was measured by Sanger sequencing. To compensate for intrinsic differences in *C*- and *G*-related fluorescence signals, sequencing electropherograms obtained with SH-SH5Y genomic DNA (Figure S3a), were used to normalize the peak areas obtained for *C*- and *G*-allele sequencing of immunoprecipitated DNA of unknown allelic composition (see Section 2. Materials and Methods).

As shown in the Sanger sequencing electropherograms (Figure 2c) and the quantification of those results (Figure 2d), the relative *C*-allele abundance (*C*%) calculated, based on data obtained with both forward and reverse sequencing primers for SH-SY5Y cells exposed to 0.01% DMSO, is roughly the same as that obtained using cellular genomic DNA (“input” = ~50%) for chromatin immunoprecipitated with anti-RXRα antibodies, and is only slightly decreased for chromatin immunoprecipitated with anti-RARα antibodies. By contrast, *C*-allele abundance is dramatically increased in chromatin immunoprecipitated with anti-RARα or anti-RXRα antibodies following atRA treatment.

Based on the observed sizes and relative abundancies of the DNA fragments in sonicated SH-SY5Y chromatin at ~200–800 bp (Figure S4a), we used ConSite online tools [30] to predict potential RARA::RXRA sites (Matrix ID: MA0159.1) [31] within a 1600 bp DNA segment centered on rs4925102 and carried out Sanger sequencing of SH-SY5Y DNA in this region to identify alleles of common SNPs, to exclude the possible influence of additional RARα/RXRα binding sites on our ChIP assays (Figure S4b). These analyses identified a single predicted RARα/RXRα binding site located 1131 bp downstream of rs4925102, with an identification threshold set at 80%, based on DNA sequence of human references genome (hg19) (Figure S4c). Because DNA fragments greater than 1 kb in length comprise only a small fraction of the sonicated chromatin used for immunoprecipitations (Figure S4a), DNA fragments containing both the rs4925102 and any upstream or downstream RARα/RXRα binding sites are unlikely to significantly contribute to the intensity of the stained PCR-amplified DNA fragments centered on the rs4925102 SNP in the ChIP experiments. The genotypes of five additional SNPs within this region in SH-SY5Y chromosomal DNA are as follows: rs117108944 (−1036, *A*/*A*), rs116249987 (−355, *A*/*A*), rs12449524 (−290, *A*/*C*), rs77893002 (−150, *AGGC*/*AGGC*), rs73292249 (+675, *C*/*C*). In addition, no indel/duplication or deletion mutations were detected in this region (see Appendix in Supplementary Materials). The haplotypes of rs12449524 and rs4925102 are *AG*/*CC*. No additional potential RARα/RXRα binding sites were identified in the sequenced SH-SY5Y genomic DNA, based on Consite online tools criteria. It should be noted that except for rs4925102, none of the genetic variants listed above are located within predicted binding motifs for RARα/RXRα heterodimers (Figure S4c). Together, these data imply that the allele-specific binding of rs4925102 by factors RARα and RXRα, as detected in the ChIP assays, is not affected by common SNPs located near rs4925102.

### 3.4. The rs4925102 DNA Region Is in Close Physical Proximity to the RAI1 Promoter in SH-SY5Y Cells with or without Exposure to atRA but Undergoes a Dramatic Change in Association with a Downstream Site within the RAI1 Gene following Exposure to atRA

SNP rs4925102 is located within the first intron of the *RAI1* gene approximately 12 Kb downstream from the mRNA transcription start site (TSS). To regulate gene expression, a remote *cis*-regulatory element is likely to interact physically with the promoter region of its cognate gene [32]. To elucidate intra-chromosome interactions of the rs4925102 locus, we employed chromatin conformation capture (3C) technology, with a DNA primer located near rs4925102 functioning as an “anchor” (A), to detect physical proximities between the rs4925102 locus and several other regions within the full extent of the *RAI1* gene, including the *RAI1* promoter region (P), in SH-SY5Y cells.

**Figure 3:**
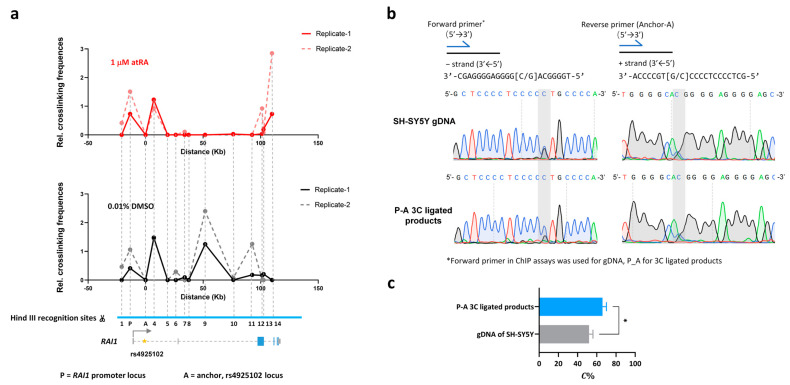
Chromatin conformation capture (3C) analysis provides evidence for significant changes in physical interactions between the rs4925102 locus and proximal and distant chromosome regions following exposure to atRA. The graphs in (**a**) show the enhanced interaction between rs4925102 locus (A) and the *RAI1* promoter (P) following a 2 h exposure to 1 μM atRA and a dramatic decrease in the interaction between this locus and a distant (50 Kb) downstream chromosome region compared to cells exposed to 0.01% DMSO. Numbers indicate positions of additional Hind III recognition sites. (**b**) Sanger sequencing electropherograms for promoter DNA segments that physically interact with the rs4925102 locus (*C* = blue traces, *G* = black traces, *T* = red traces and *A* = green traces). (**c**) Quantitative results for sequencing peak plots. *C*% represents the proportion of positive sequence *C* in the linkage products (*: *p* < 0.05; based on Student’s *t*-test).

As shown in Figure 3a, 3C analysis detected physical proximity between the rs4925102 locus and the upstream *RAI1* promoter in SH-SY5Y cells, following exposure to 0.01% DMSO or 1 μM atRA. After 3C detection, Sanger sequencing was used to measure the relative *C*-allele abundance of rs4925102 in the 3C ligated products (P-A) containing the rs4925102 locus and the upstream *RAI1* promoter in SH-SY5Y cells exposed to 1 μM atRA (Figure 3b). The areas under the *C*- and *G*-traces for the rs4925102 polymorphism in the sequencing electropherogram were measured using Image J software and normalized using SH-SY5Y genomic DNA as described in Section 2. Materials and Methods. The quantitative data were statistically tested using Student’s *t*-test. These results revealed that the rs4925102 *C*-allele was present at significantly higher levels than the G-allele within the ligated products (P-A) (Figure 3c).

In addition to physical interaction between the rs4925102 locus and *RAI1* promoter, 3C analysis revealed a highly robust loss of an interaction between the rs4925102 locus and a chromosomal site located ~50 kb downstream of rs4925102 following exposure to atRA treatment compared to the vehicle control. This result strongly suggests that exposure to atRA induces a major change in the topology of the *RAI1* gene. The role of this RA-induced change in the conformation of the *RAI1* gene in *RAI1* gene expression and function of *RAI1* during development and within the mature brain remains a topic for future investigation.

### 3.5. Autism Spectrum Disorder (ASD) Case-Control and Transmission Disequilibrium Test (TDT) Studies of rs4925102

Because changes in *RAI1* expression are associated with autistic behaviors in both SMS and PTLS syndromes, we investigated the possible role of rs4925102 in ASD. As shown in Table 1, we found a statistically significant correlation between rs4925102 genotypes and diagnosis of ASD in a case-control study, and a near statistically significant correlation with ASD in a TDT study using Caucasian subjects.

## 4. Discussion

In the present study, we investigated the regulatory activity of the *RAI1* rs4925102 locus in the human cell lines HEK-293 and SH-SY5Y. We first demonstrated that inserting rs4925102 closely upstream from a minimal promoter increases the expression of a luciferase reporter gene 24 h after transient transfection into the above cells (Figure 1a,b). A search of the RegulomeDB (using hg19 as the reference genome), revealed that this SNP is located within predicted binding motifs of the transcription factors SP1, SP4, and RARα/RXRα heterodimers (Figure S1). Based on these observations, we predicted that *RAI1* expression could be induced by the RARα/RXRα heterodimer agonist, all-*trans* retinoic acid (atRA). Consistent with this prediction, exposure to 1 μM atRA increased luciferase reporter expression in SH-SY5Y cells transfected with our enhancer construct, with the highest levels of induction observed for rs4925102 *C*-allele (Figure 1c).

To obtain additional insights into allele-specific activation of *RAI1* expression mediated by rs4925102, we carried out chromosome immunoprecipitation (ChIP) experiments to quantify levels of RARα/RXRα binding to endogenous rs4925105 in neuronal SH-SY5Y cells with or without exposure to 1 μM atRA (Figure 2). Our observation that RARα/RXRα preferentially binds the rs4925102(C) locus compared to the rs4925102(G) locus after exposure to atRA, may explain at least in part the preferential activation of reporter gene expression by rs4925102(C) following exposure to atRA. Consistent with these observations, we also found that the rs4925102 locus associates with the *RAI1* promoter, with a preference for the rs4925102(C) allele (Figure 3).

We initially found it difficult to explain the increased expression of the reporter gene in the absence of exposure to atRA (Figure 1). The results of ChIP experiments showed, however, that exposure to atRA results in a large increase in RARα/RXRα bound to the rs4925102 locus, suggesting that this site may be occupied by other activating transcription factors in the absence of exposure to atRA. Consistent with this possibility, the rs4925102 locus is predicted to bind the transcription factors SP1, SP4, and ZNK148 (Figure S1). Identifying transcription factors that bind to the rs4925102 in the absence of atRA exposure will be explored in future studies.

A surprising and important finding in this study is the dramatic change in the association of the rs4925102 locus with a chromosomal site located ~50 Kb downstream following exposure to atRA, as revealed in our 3C experiments (Figure 3). A straight-forward interpretation of this observation is that exposure to atRA induces a long-distance change in the physical configuration, i.e., the topology, of the *RAI1* gene. In fact, atRA-induced remodeling of presumptive three-dimensional chromatin loops has previously been reported for RA-regulated genes, including *CASP9* and *CYP26A1* [33]. Correlations between extracellular stimulation-promoted changes in gene expression and topological changes in those genes have been observed for other nuclear receptors [34,35,36,37] and are in agreement with the general observation that gene expression and chromosome architecture are closely interdependent and influence each other, especially in the case of long-distance enhancers [32,38]. Elucidating the roles of atRA-induced changes in *RAI1* expression during early brain development and in the mature brain remains an important goal for subsequent studies.

Finally, previous studies demonstrated that RA signaling is important for both neuronal-development [39,40,41] and adult brain function [20,39]. In particular, the observations that both deletions or duplications of the human *RAI1* are associated with brain pathologies, including sleep disorders and autistic behaviors, suggest that *RAI1* is a dosage-sensitive gene where 50% higher or lower changes in gene expression are sufficient to produce pathological changes in brain development and function. Based on these observations, it is plausible that modest changes in *RAI1* expression also contribute alone or in combination with other genes to ASD and other brain disorders.

As a step toward investigating this possibility, we examined statistical correlations between rs4925102 genotypes and the diagnosis of autism spectrum disorder (ASD) with samples from a case-control association study [26] and transmission disequilibrium test (TDT) [25] in the Caucasian population. As shown in Table 1, these studies yield a nominally significant correlation of G/C odds ratios in the case-control study and a near-significance correlation in the TCT study, with the “low-expression” *G*-allele identified as the risk allele in both studies. While not conclusive by themselves, these results suggest that the rs4925102 enhancer plays an important role in regulating *RAI1* functions in the brain. Future genetic studies will examine how alleles of this enhancer interact with regulatory alleles of genes that function upstream and downstream of *RAI1* in neuronal cells in the developing and mature brain.

## Figures and Tables

**Table 1:** Correlations between SNP rs4925102 genotype and ASD diagnosis in TDT tests and a case-control study in Caucasian populations.

Test	Sample Size	Minor/Major Allele	OR * (95% Cl)	*p*-Value	Risk Allele
TDT	4076	G/C	1.119 (0.997–1.256)	0.056	G
Case-control	2477	G/C	1.123 (1.002–1.258)	0.046	G

* OR = odds ratio defined with respect to minor allele; 95% CI = 95% confidence interval.

## Data Availability

The original contributions presented in the study are included in the article/Supplementary Material.

## Supplementary Materials

The following supporting information can be downloaded at: https://www.mdpi.com/article/10.3390/genes15040460/s1, Figure S1: Location and chromosomal context of the regulatory variant rs4925102 in human *RAI1* intron 1; Figure S2: All-*trans* retinoic acid induces transactivation of *RAI1* in human neuroblastoma cell line, SH-SY5Y cells in dose-dependent manner; Figure S3: Calculation of ***A***_forward_ and ***A***_reverse_ correction factors based on Sanger sequencing of SH-SY5Y genome DNA; Figure S4: Chromatin immunoprecipitation (ChIP) experiments using SH-SY5Y cells provide evidence for the binding of RARα/RXRα to chromosomal fragments containing SNP rs4925102; Figure S5: Hind III recognition sites within the human *RAI1* locus; Table S1: Primers used in plasmid constructions; Table S2: Primers used in chromatin conformation capture (3C) assays; Appendix: selected DNA sequencing results of SH-SY5Y cells.
