# Supplementary material for: Allele-Specific Regulation of the Candidate Autism Liability Gene RAI1 by the Enhancer Variant rs4925102 (C/G)
Source: Genes (Basel). 2024 Apr 6;15(4):460. doi: 10.3390/genes15040460 (PMC11049881; doi:10.3390/genes15040460)
Supplement: Supplementary file 1 [file genes-15-00460-s001.zip › genes-2941058-supplementary.pdf]

Supplementary Materials for

Allele-specific Regulation of the Candidate Autism Liability Gene *RAI1* by the Enhancer Variant rs4925102 (*C*/*G*)

Xi Yuan [1], Li Chen [2,*] and David Saffen [1,2,3,*]

[1] Institutes of Brain Science, Fudan University, Shanghai 200032, China; xyuan9209@163.com
[2] Department of Cellular and Genetic Medicine, School of Basic Medical Sciences, Fudan University, Shanghai 200032, China;
[3] State Key Laboratory of Medical Neurobiology, Fudan University, Shanghai 200032, China;
* Correspondence: chenli2008@fudan.edu.cn (L.C.); saffen@fudan.edu.cn or david.saffen2@gmail.com (D.S.)

This PDF file includes:

Figs. S1 to S5
Tables S1 to S2
Appendix

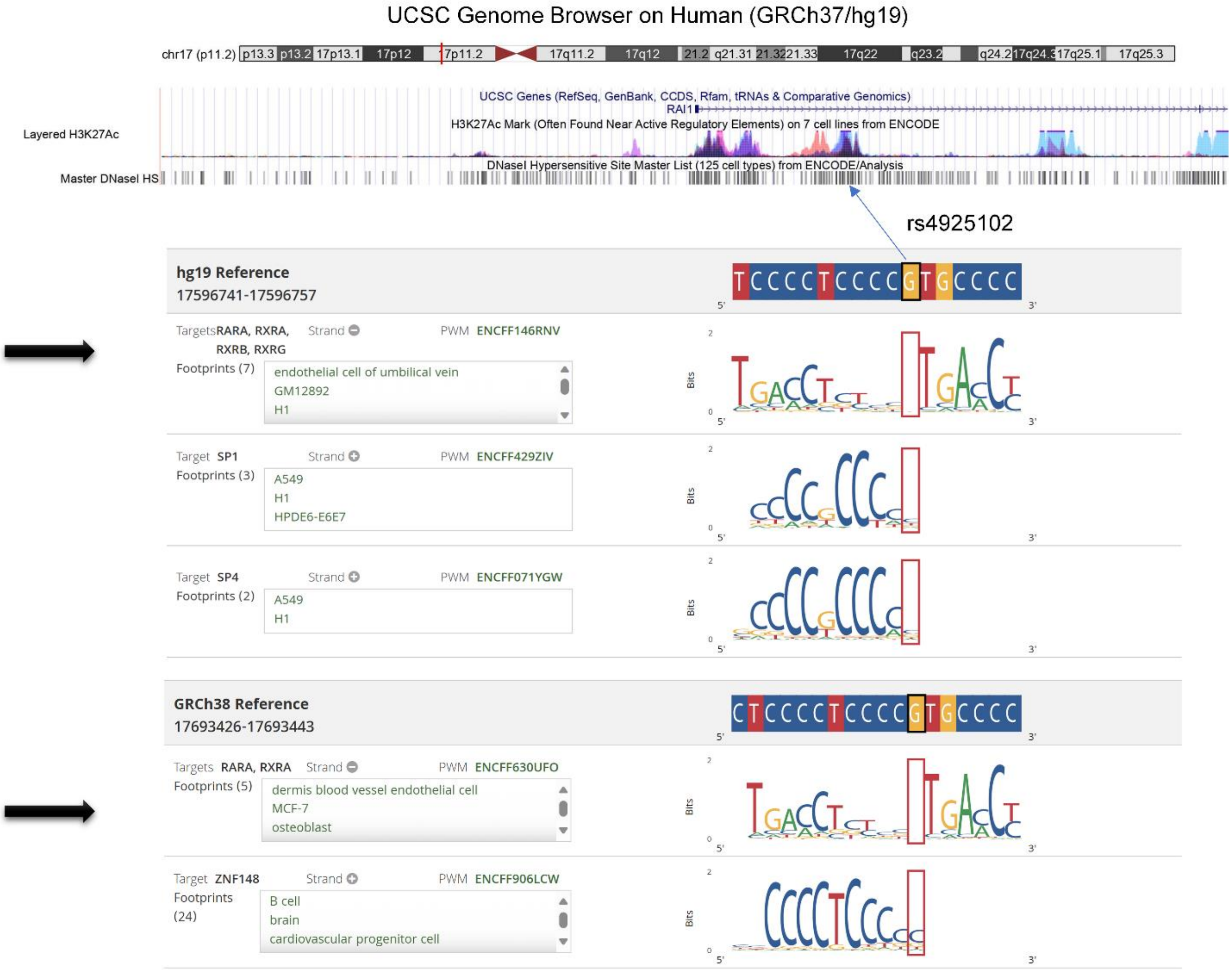

Figure S1. Location and chromosomal context of the regulatory variant rs4925102 in human *RAI1* intron 1. (**top**) Screen shot from the UCSC Genome Browser for the indicated region of Chromosome 17 showing the positions of the *RAI1* gene and histone III lysine 27 acetylation- and DNase sensitive-clusters. (**bottom**) Screen shots from RegulomeDB database (http://www.regulomedb.org/) based on hg19 and GRCh38 versions of the reference genome showing the locations of SNP rs4925102, RARα/RXRα, SP1, SP4, and ZNF148 consensus binding sequences and DNA sequences protected by the listed transcription factors in DNase footprint assays.

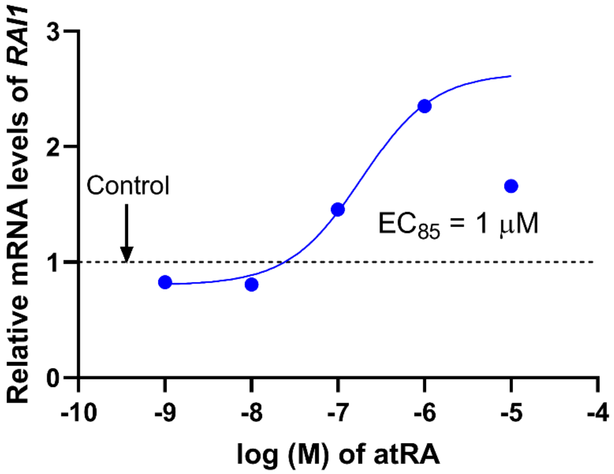

Figure S2. All-*trans* retinoic acid (atRA) increases *RAI1* mRNA expression in the human neuroblastoma cell line SH-SY5Y in dose-dependent manner. SH-SY5Y cells were exposed to increasing concentrations (1 nM, 10 nM, 100 nM, 1 µM, and 10 µM) of atRA or 0.01% dimethylsulfoxide (DMSO) carrier solvent alone (control) for 24 hrs. The measured *RAI1* mRNA levels were normalized to measured level of the house-keeping gene *GAPDH* mRNA in each sample. Relative mRNA levels of *RAI1* are listed as fold-increase compared to the level measured in control SH-SY5Y cells exposed to DMSO alone. Nonlinear regression analysis was carried out using GraphPad Prism (v9.0), which predicted an effective concentration of EC_{85} for ~1 µM RA.

Methods: Total RNA was isolated using TRIzol reagent (Invitrogen, Carlsbad, MA, USA). Reverse transcription was performed using the PrimeScript™ II 1st Strand cDNA Synthesis kit (TaKaRa, Tokyo, Japan). The level of target mRNA was normalized to the level of *GAPDH*. Real-time quantitative PCR was performed using SYBR® Green Realtime PCR Master Mix (TOYOBO, Kita-ku, Osaka, Japan). The PCR primer sequences are as follows, *RAI1*: 5′-AGACCCAGGAGGAGCCC-3′ (forward), 5′-TGCGAGGTCTGCTGGTAGTT-3′ (reverse); *GAPDH*: 5′-TCAAGATCATCAGCAATGCC-3′ (forward), 5′-TGTGGTCATGAGTCCTTCCA-3′ (reverse).

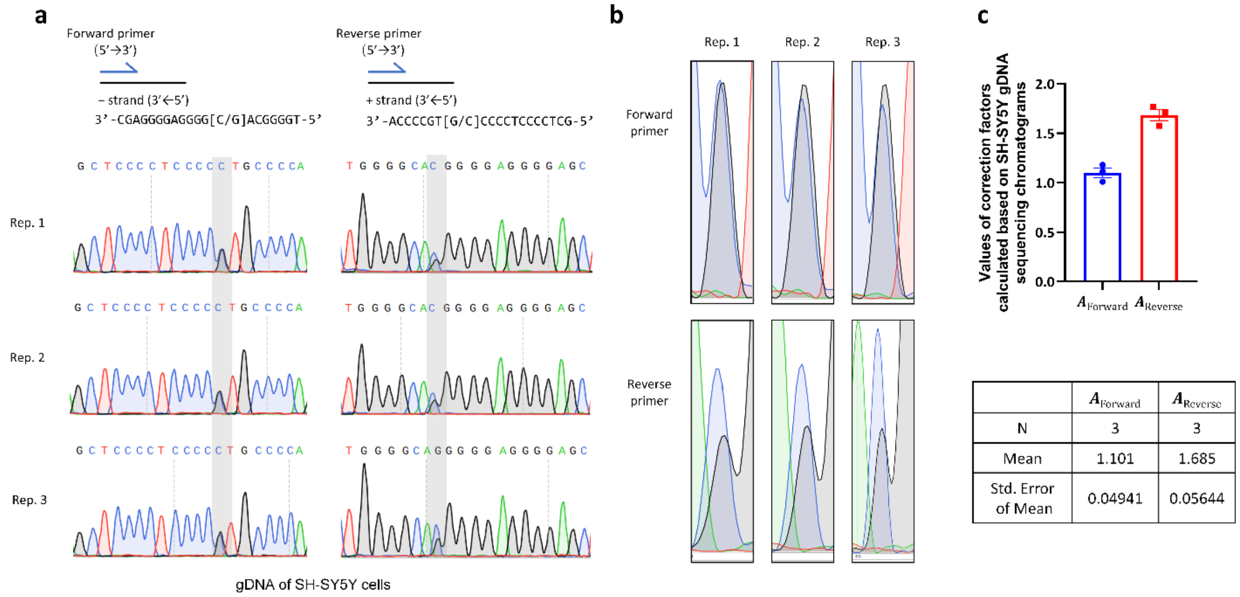

	A_{Forward}	A_{Reverse}
N	3	3
Mean	1.101	1.685
Std. Error of Mean	0.04941	0.05644

Figure S3. Calculation of A_{forward} and A_{reverse} correction factors based on Sanger sequencing of SH-SY5Y genome DNA. To normalize ratios of peak areas for rs4925102 *C*- and *G*-alleles in Sanger sequencing electropherograms obtained using immunoprecipitated DNA of unknown allelic composition, intrinsic differences in *C*- and *G*-allele-related fluorescence signals were quantified using DNA extracted from SH-SH5Y cells, which are heterozygous for rs4924102(*C*/*G*). (**a**) Sanger sequencing electropherograms obtained for SH-SY5Y genomic DNA (gDNA) using forward or reverse sequencing primers described in the Methods section of the main text. (**b**) Enlarged screenshots of overlapping rs4925102-*C* and -G peaks in the electropherograms shown in panel (**a**). (**c**) Values of $\boldsymbol{A}_{\text{Forward}}$ and $\boldsymbol{A}_{\text{Reverse}}$ correction factors calculated based on three independent experiments and listed as mean ± its standard error (SEM).

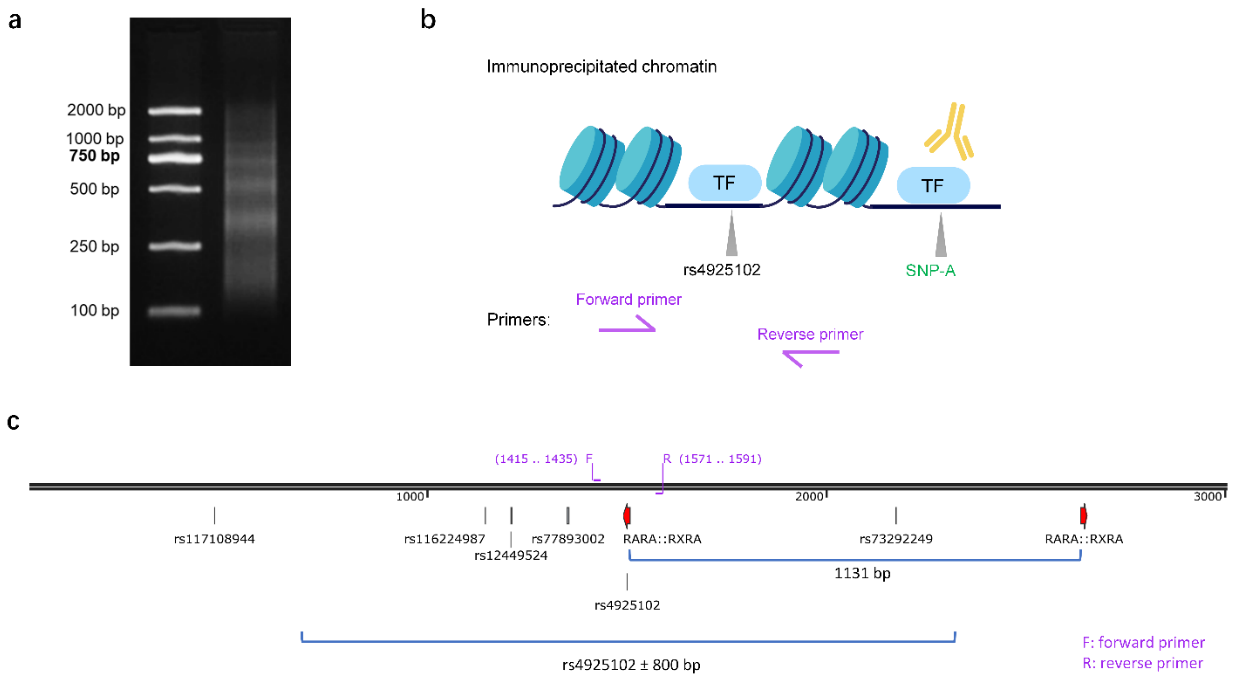

Figure S4. Chromatin immunoprecipitation (ChIP) experiments using SH-SY5Y cells provide evidence for the binding of RARα/RXRα to chromosomal fragments containing SNP rs4925102. (**a**) Agarose gel electrophoresis of DNA purified from sonicated chromatin, revealed DNA fragments ranging in size from ~300 to ~1000 bp. Similar results were obtained in replication experiments (*not shown*). (**b**) A schematic diagram showing how false-positive signal could be produced by a nearby TF binding site that contains a SNP- A that is in linkage disequilibrium (LD) with rs4925102. If the genotype of SNP-A influences the binding of TFs (e.g., RARα/RXRα) with which it interacts, the binding-favorable-genotype of SNP-A as well as the genotype of rs4925102 that is in LD with SNP-A would be enriched in the immunoprecipitated chromatin. (**c**) Locations of RARα/RXRα binding sites (RARA::RXRA) predicted using JASPAR online tool (jaspar.elixir.no) within a chromosomal segment spanning rs4925102 ± 1500 bp. Predicted binding threshold for the RARα/RXRα dimer (red) was 80%. Also shown are the locations of common SNPs and forward (F) and reverse (R) PCR primers used to detect DNA segments that include rs4925102 (purple). DNA fragments containing both the rs4925102 and the upstream or downstream RARA::RXRA sites are unlikely to significantly contribute to the intensity of the stained PCR-amplified DNA fragments centered on the rs4925102 SNP in the ChIP experiments, as DNA fragments over 1 kb only make up a small fraction of the chromatin used for immunoprecipitation (based on the gel shown in panel (**a**)).

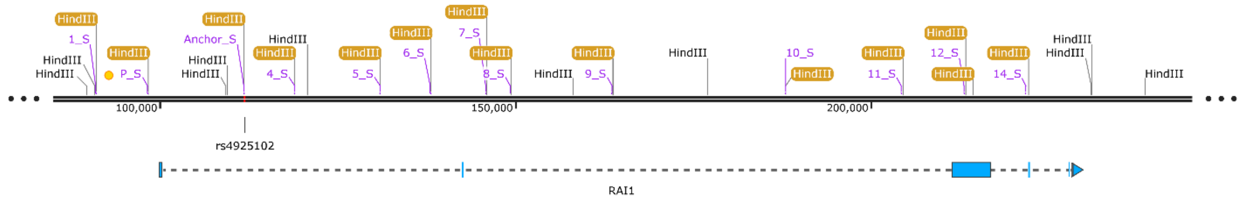

Figure S5. *Hin*d III recognition sites within the human *RAI1* locus. The *Hin*d III recognition sites marked with orange backgrounds were included in the control template for 3C-qPCR. Primers represented in purple font are the forward primers of primer pairs used to amplify PCR products covering each targeted *Hin*d III recognition site. Constructions prepared with these PCR products were pooled in equal molar amounts and digested with *Hin*d III followed by ligation to form the control template used in 3C-qPCR detections.

Table S1. Primers used in plasmid constructions.

Oligo Name	Allele	Sense or Anti-sense	Sequence (5′-3′)*
C-rs49-S	C	Sense	**TCGAG** CCTCTCCAGCTCCCCTCCCC**C**TGCCCCAGGCATCTTT
C-rs49-A	C	Anti-sense	**AGCT** AAAGATGCCTGGGGCA**G**GGGGAGGGGAGCTGGAGAGG **C**
G-rs49-S	G	Sense	**TCGAG** CCTCTCCAGCTCCCCTCCCC**G**TGCCCCAGGCATCTTT
G-rs49-A	G	Anti-sense	**AGCT** AAAGATGCCTGGGGCA**C**GGGGAGGGGAGCTGGAGAGG **C**

*Underlined bases indicate SNP alleles and sequences in **BOLD** type indicate restriction endonuclease recognition sites.

Table S2. Primers used in chromatin conformation capture (3C) assays.

Primers	Sequences (5′-3′)
*RAI1*_exon3_S	ATAACCAGCCCGAGTCATGCA
*RAI1*_exon3_A	TGATGTTTCCTGCGAGGTCTG
1_S	TTGGGTGTGACTGATATTCTCCG
1_A	AACTTTCCCACAATTAGAGC
P_S	TGTCATCCAGAAACCGCACT
P_A	GTCTCCCAGGGTGAATGTCAA
Anchor-S	GGGCAGCCTTCCTGATTGAC
Anchor-A	CGCAGCATCTTAGAATGTAGG
4_S	CATCAAAGCGTCTTCTCGTT
4_A	GGGCGTCGTTTTCCAGGTAA
5_S	AGGTCAAATGGCATAGGAGA
5_A	CCCACTCAGCCATTACAACA
6_S	TTCCATAGCACGCTTCACCTA
6_A	AAGGTGGCAAAAGTATGCATC
7_S	GACCCAGCTACTGATTTGCC
7_A	TCCCGTGAACACACCCAGTCG
8_S	CGAGGCACAAGATGAGACTT
8_A	ACACCCACAGAAGAGTGACA

9_S	GCCTGTAAAGTGATGATGGAA
9_A	CCCTTTCCTTAGAATATGCTC
10_S	CGTCATAATGAAGGAGCGACA
10_A	TACCCACCAGAAATTCCATGC
11_S	GATCCCCTTTGCATCGGTTC
11_A	ATCCTGCTCCCAAATAAAGTGG
12_S	ACATGTCCACCAAATCTGACGA
12_A	GTGGCTTGCTGCTGTCCTTG
13_S	TGGCCCAGAAGCCCAACAAGC
13_A	CCGTGAGAGAACGAGTACACAT
14_S	TGTGTACCCCAGTGCTGAAC
14_A	AGGTGTGGAGGCATCCTTTG

Appendix:

Notes:

The genotypes of five additional SNPs within this region in SH-SY5Y chromosomal DNA are as follows: rs117108944 (−1036, *A*/*A*), rs116249987 (−355, *A*/*A*), rs12449524 (−290, *A*/*C*), rs77893002 (−150, *AGGC*/*AGGC*), rs73292249 (+675, *C*/*C*). In addition, no indel/duplication or deletion mutations were detected in this region. The haplotypes of rs12449524 and rs4925102 are *AG*/*CC*.

6 common variants were highlighted with different colors as rs117108944, rs116249987, rs12449524, rs77893002, rs4925102, and rs73292249.

>Selected DNA sequencing results of genotype of SH-SY5Y with rs4925102 (*G*)
hg19_dna range=chr17:17595022-17597816 5'pad=0 3'pad=0 strand=+
AGGCAAAAGAGAGGACTAAACCCTTTAGTCTGTTTGAGGGAACTCCAGCC
CCCAGCATGTTGCTCTCTGGGGGATCCAGCAGGGCCAGGTGGGCAGTGGG
AGCACCCTAGCCCCAGGTGCTACCTTCCAGCCTGGGGCCAAGCCTTTTCC
CTTTCTCTCATTTGCAGCTTTCTGATAGGTGGGAAAGAGAAGCAGGAAAA
AAGAGCAGAGGAAAAACAATATTTTCATTTCAAACACAGATAGGGGTGAG
GGAGGCGGGAAGTAATCTGGTTTATGATACCTTTGAGACAAGCACAGAAA
ATCCCCACCGTGGCCCGAAATAGCACTAAAACAGACGTTTTGCCAAATCA
GACTTAATTGAGTGGCTCTCTTCTTGACTCCAGCAGGATGTGGCTGGAGC
TGGCTTTGTTCCCTTGGGGTGACACTGTCCCCTGGGGCCCCTGCCCTGGC
TAATTAAACAAAGAGGACCTGGTCTCCTAGGCAGGACAGGAGCCTGGGGA
GCTGAACTTGACAGCCCTTGGCCTCTGCCTGAGAGGGCCAGAAATGGTTA
CAGGGGTTGGGAAGGTGGGGGGTTCCTGACAGGAGGGCCAACTGAGTGAA
AGCATTGGTGGGAGAGGCCTGGTTCCTCTAATATGTGAATATCTGTCTCT
TTGGATGCTGAAAGGGATGGAGAAAAGCCAGCATTGAGTGCCTACTATTG
TGCCAGGCATTGGGTGGTCCTCGCTAAGTCTTATTAACTGCCCTATATCC
ATTATCAAGGAAACTGAAGCACAGAGAGGTTAAGTGATCTGCCTGAGGTC
ACACAGCAACCAAGTGGCAGAGCTAGGATTAGAAATCACATCCGCAGGCT
CCAAAGCCAGGCCCTGCCTGCTGCTTACCCCCTTCCCCATGTTAGTCACT
CATCCATTCGGGAGCTAATTCCTGGCTATGAAATAATATTGTGACCTTGT
GGGAAGTGAAGGCTTGCAGTCAGACAGCCATGAGCTTGGCTTCTGACCAA
AGCAGTTTTTCTTTTCATGGTTTTCTCCCTCAGAACCTCCTCTTTCCTCA
TCTATCAAGTGGGCATAATCATGCCTTTATCCTGGAGTTGCTGTAAGGGT
TAAATTCAGCACAGAAAGGGATGGAAGGGGAAGGAGGAGGTCCTTGGGCC
ACCCAGCTTGGCAGGGTGCAATAAAAGAGTCCTAGGTAGAGGGAGCCAGG
CAAGATTGCAATATCTCTGTGCCTATCAGTGTTGGGTTTCTTTATTTGAA
AGGGGAGAGAGGGCAGACTCCACTCTGCAGGTAGAGAAGGTGGTGTGGAA
TGCGGGTATTTATTTTTCTTTGCCTGTGAAAGCCCTGTTTTCGCTCAGGC

TGTGTAGGGCTTCCCGACACTGCCATGGGTGCGCACATCTGCACACTCTG
GGGTGCCTGCCATGCAGAGGCATCTGCCCAGGGTGGGCAATGAGGGCATC
CACCATGTGACGCCCCCGTGCAGCCTGTAAGCTCTCATCACAGTGCCTGG
GATACCTGGGAGTCCCCAGAAAGAGTGAGGATGGGTGAGGGCCATGAAAC
CAGGGCATCATTCCTCCACCTCCTCTGATCAGGCCCTCCTGGTCCCATTC
ATCTCCTTATCTCCTGGCAGCCAGTGGGTTGCTGGGAAGGCAGGGGCAGC
CTTCCTGATTGACAAACAAAACAAGCTTGATCACAAAAGGAGAGGGGAGA
CTGGCTGGGCCTCTCCAGCTCCCCTCCCCGTGCCCCAGGCATCTTTCTTG
CTCCACCCAACTGGCACCCTACATTCTAAGATGCTGCGAGGGGGCTCCAA
AGCCATTGTCGAAGGTCAGGCCACCCAGGCTAGGAGCCCCTTGCACTGGG
GAGGTGGATTCAAGGAGTGATGGGGTTGCCATGAGGCGGCGCTTGCTGCA
ATGGCCGCCCCTCCTTCCCTTCAGCTGGCTGGGCAGGCAGCTTGGAGAGG
AAGCCAGGCGGCAGGTAGCTGAGAAAGACATCGGATCCAGGGGGAGCAGC
AGAGAAAGCAAACAAACAGGGCTTTGTGTGGCTGCAGATTGGCGGCTCAG
AAGGGAAGCCAGGCCTGTGGTAGGGGGAAGGGAGAGGAAGGAACGGGAGA
CCAGGCCATTGCTCTGGGCCAGAGATCGGCGGCTGGACCAGCCCGGGCTG
GGCGGCCGGGAAGTAGCGAGTGCCACTACGTGTGGACAGGCATCTGGAGT
CACAGCCTGGCCGAGTCCCAGCTCTGCCACTTCACTCCTTAACAGATCTG
GGCTTGTTTGCCCACCTAATCTGGGCAGTCATTCACACGTAGGACTCGCT
GGCTTAGGAGGAGAGTCACCTGTCCTGGCCCAGTCAGTGTCTGCTCTCAT
CCTACCCTTGTCCCGTCATTGCTGGCAGGACACAGAATCAAAGGGATTCA
GGGTCTCCTGGGAGCTGCTAGCCTTGCCGGGTCTCCTGGGAGGGCAGCGA
CTTCTGCTTAGATCTGTGGGCACTTTGGAGTCTTTGACCGGGGAGGCAGC
CGTGCTGAGGGGAAACGGGTTCTCTGCCCAATAACGGATGCCTGGGGTGT
AGGTGGGCAGAGGCTGGCATCAGGCCTGAGGAACCAAACTGCCCCCTCCT
CGTCGCGGTGGAGACGCACGAGGTGCGGGGAGGAACATAGCCAGGAAGAC
GGGCCTTCCTGTGGGGAGGGAGGGGCATCTTTTCTGGGGTGTGAGTCACA
TTCTGGATCCCAGCGAGGGCAGCCCCCAACCTGGCCCACCTCTCAGAGGG
CTGGGAGGGCCCGGGCCTCGAGGAAAGGCATAGGTAGGAGCTGTC

>Selected DNA sequencing results of genotype of SH-SY5Y with rs4925102 (C)
hg19_dna range=chr17:17595022-17597816 5'pad=0 3'pad=0 strand=+
AGGCAAAAGAGAGGACTAAACCCTTTAGTCTGTTTGAGGGAACTCCAGCC
CCCAGCATGTTGCTCTCTGGGGGATCCAGCAGGGCCAGGTGGGCAGTGGG
AGCACCCTAGCCCCAGGTGCTACCTTCCAGCCTGGGGCCAAGCCTTTTCC
CTTTCTCTCATTTGCAGCTTTCTGATAGGTGGGAAAGAGAAGCAGGAAAA
AAGAGCAGAGGAAAAACAATATTTTCATTTCAAACACAGATAGGGGTGAG
GGAGGCGGGAAGTAATCTGGTTTATGATACCTTTGAGACAAGCACAGAAA
ATCCCCACCGTGGCCCGAAATAGCACTAAAACAGACGTTTTGCCAAATCA
GACTTAATTGAGTGGCTCTCTTCTTGACTCCAGCAGGATGTGGCTGGAGC
TGGCTTTGTTCCCTTGGGGTGACACTGTCCCCTGGGGCCCCTGCCCTGGC
TAATTAAACAAAGAGGACCTGGTCTCCTAGGCAGGACAGGAGCCTGGGGA
GCTGAACTTGACAGCCCTTGGCCTCTGCCTGAGAGGGCCAGAAATGGTTA
CAGGGGTTGGGAAGGTGGGGGGTTCCTGACAGGAGGGCCAACTGAGTGAA
AGCATTGGTGGGAGAGGCCTGGTTCCTCTAATATGTGAATATCTGTCTCT
TTGGATGCTGAAAGGGATGGAGAAAAGCCAGCATTGAGTGCCTACTATTG
TGCCAGGCATTGGGTGGTCCTCGCTAAGTCTTATTAACTGCCCTATATCC
ATTATCAAGGAAACTGAAGCACAGAGAGGTTAAGTGATCTGCCTGAGGTC
ACACAGCAACCAAGTGGCAGAGCTAGGATTAGAAATCACATCCGCAGGCT
CCAAAGCCAGGCCCTGCCTGCTGCTTACCCCCTTCCCCATGTTAGTCACT
CATCCATTCGGGAGCTAATTCCTGGCTATGAAATAATATTGTGACCTTGT
GGGAAGTGAAGGCTTGCAGTCAGACAGCCATGAGCTTGGCTTCTGACCAA
AGCAGTTTTTCTTTTCATGGTTTTCTCCCTCAGAACCTCCTCTTTCCTCA
TCTATCAAGTGGGCATAATCATGCCTTTATCCTGGAGTTGCTGTAAGGGT
TAAATTCAGCACAGAAAGGGATGGAAGGGGAAGGAGGAGGTCCTTGGGCC
ACCCAGCTTGGCAGGGTGCAATAAAAGAGTCCTAGGTAGAGGGAGCCAGG
CAAGATTGCAATATCTCTGTGCCTATCAGTGTTGGGTTTCTTTATTTGAA
AGGGGAGAGAGGGCAGACTCCACTCTGCAGGTAGAGAAGGTGGTGTGGAA
TGCGGGTATTTATTTTTCTTTGCCTGTGAAAGCCCTGTTTTCGCTCAGGC
TGTGTAGGGCTTCCCGACACTGCCATGGGTGCGCACATCTGCACACTCTG
GGGTGCCTGCCATGCAGAGGCATCTGCCCAGGGTGGGCACTGAGGGCATC
CACCATGTGACGCCCCCGTGCAGCCTGTAAGCTCTCATCACAGTGCCTGG
GATACCTGGGAGTCCCCAGAAAGAGTGAGGATGGGTGAGGGCCATGAAAC
CAGGGCATCATTCCTCCACCTCCTCTGATCAGGCCCTCCTGGTCCCATTC
ATCTCCTTATCTCCTGGCAGCCAGTGGGTTGCTGGGAAGGCAGGGGCAGC
CTTCCTGATTGACAAACAAAACAAGCTTGATCACAAAAGGAGAGGGGAGA
CTGGCTGGGCCTCTCCAGCTCCCCTCCCCCTGCCCCAGGCATCTTTCTTG
CTCCACCCAACTGGCACCCTACATTCTAAGATGCTGCGAGGGGGCTCCAA
AGCCATTGTCGAAGGTCAGGCCACCCAGGCTAGGAGCCCCTTGCACTGGG
GAGGTGGATTCAAGGAGTGATGGGGTTGCCATGAGGCGGCGCTTGCTGCA
ATGGCCGCCCCTCCTTCCCTTCAGCTGGCTGGGCAGGCAGCTTGGAGAGG

AAGCCAGGCGGCAGGTAGCTGAGAAAGACATCGGATCCAGGGGGAGCAGC
AGAGAAAGCAAACAAACAGGGCTTTGTGTGGCTGCAGATTGGCGGCTCAG
AAGGGAAGCCAGGCCTGTGGTAGGGGGAAGGGAGAGGAAGGAACGGGAGA
CCAGGCCATTGCTCTGGGCCAGAGATCGGCGGCTGGACCAGCCCGGGCTG
GGCGGCCGGGAAGTAGCGAGTGCCACTACGTGTGGACAGGCATCTGGAGT
CACAGCCTGGCCGAGTCCCAGCTCTGCCACTTCACTCCTTAACAGATCTG
GGCTTGTTTGCCCACCTAATCTGGGCAGTCATTCACACGTAGGACTCGCT
GGCTTAGGAGGAGAGTCACCTGTCCTGGCCCAGTCAGTGTCTGCTCTCAT
CCTACCCTTGTCCCGTCATTGCTGGCAGGACACAGAATCAAAGGGATTCA
GGGTCTCCTGGGAGCTGCTAGCCTTGCCGGGTCTCCTGGGAGGGCAGCGA
CTTCTGCTTAGATCTGTGGGCACTTTGGAGTCTTTGACCGGGGAGGCAGC
CGTGCTGAGGGGAAACGGGTTCTCTGCCCAATAACGGATGCCTGGGGTGT
AGGTGGGCAGAGGCTGGCATCAGGCCTGAGGAACCAAACTGCCCCCTCCT
CGTCGCGGTGGAGACGCACGAGGTGCGGGGAGGAACATAGCCAGGAAGAC
GGGCCTTCCTGTGGGGAGGGAGGGGCATCTTTTCTGGGGTGTGAGTCACA
TTCTGGATCCCAGCGAGGGCAGCCCCCAACCTGGCCCACCTCTCAGAGGG
CTGGGAGGGCCCGGGCCTCGAGGAAAGGCATAGGTAGGAGCTGTC
